# Contrasted life trajectories: reconstituting the main population exposomes in French Guiana

**DOI:** 10.3389/fpubh.2023.1247310

**Published:** 2024-01-11

**Authors:** Mathieu Nacher, Célia Basurko, Maylis Douine, Yann Lambert, Cyril Rousseau, Celine Michaud, Ronan Garlantezec, Antoine Adenis, Margarete M Gomes, Kinan Drak Alsibai, Nadia Sabbah, Véronique Lambert, Loïc Epelboin, Rakesh Gajadhar Sukul, Fredrik Terlutter, Caroline Janvier, Najeh Hcini

**Affiliations:** ^1^CIC INSERM, Centre Hospitalier de Cayenne, Cayenne, French Guiana; ^2^Université de Guyane, Cayenne, French Guiana; ^3^Amazonian Infrastructures for Population Health, Cayenne, French Guiana; ^4^Centres délocalisés de Prévention et de Soins, Centre hospitalier de Cayenne, Cayenne, French Guiana; Département Recherche Innovation Santé Publique, Cayenne, French Guiana; ^5^Épidémiologie et science de l’exposition en santé-environnement (Elixir), Institut de Recherche en Santé Environnement et Travail (IRSET), Rennes, France; ^6^Santé publique et épidémiologie, CHU de Rennes, Rennes, France; ^7^Superintendencia de vigilancia em Saude do Amapa, Macapa, Brazil; ^8^Centre de Ressources Biologiques Amazonie, Centre Hospitalier de Cayenne, Cayenne, French Guiana; ^9^Service d’endocrinologie diabétologie, Centre hospitalier de Cayenne, Cayenne, French Guiana; ^10^Western French Guiana Hospital, Saint Laurent du Maroni, French Guiana; ^11^Service des Maladies Infectieuses et Tropicales, Centre hospitalier de Cayenne, Cayenne, French Guiana; ^12^Director of Health, Ministry of Health, Paramaribo, Suriname; ^13^Service de Psychiatrie, Centre hospitalier de Cayenne, Cayenne, French Guiana

**Keywords:** exposome, nutritional deficiencies, infectious diseases, heavy metals, French Guiana, life statistics, obesity, health inequalities

## Abstract

In French Guiana, life expectancy is between 2 and 3 years below that of France, reflecting differences in mortality rates that are largely sensitive to primary healthcare and thus preventable. However, because poverty affects half of the population in French Guiana, global measurements of life expectancy presumably conflate at least two distinct situations: persons who have similar life expectancies as in mainland France and persons living in precariousness who have far greater mortality rates than their wealthier counterparts. We thus aimed to synthesize what is known about statistical regularities regarding exposures and sketch typical French Guiana exposomes in relation to health outcomes. We conducted a narrative review on common exposures in French Guiana and made comparisons between French Guiana and mainland France, between rich and poor in French Guiana, and between urban and rural areas within French Guiana. The most striking fact this panorama shows is that being a fetus or a young child in French Guiana is fraught with multiple threats. In French Guiana, poverty and poor pregnancy follow-up; renouncing healthcare; wide variety of infectious diseases; very high prevalence of food insecurity; psychosocial stress; micronutrient deficiencies; obesity and metabolic problems; and frequent exposure to lead and mercury in rural areas constitute a stunningly challenging exposome for a new human being to develop into. A substantial part of the population’s health is hence affected by poverty and its sources of nutrition.

## Introduction

The exposome concept aimed to focus attention on the need for an integrated non-genetic environmental exposure assessment in epidemiological studies (1–3). The disease often results from the combination of particular environmental exposures operating on a particular genetic background (4). While individual disease susceptibility was explored through genome-wide association studies throughout the world, there was no comparable effort for non-genetic factors. This is a problem as epidemiology aims to reveal unknown effects associated with specific environmental exposures. Through the detailed description of lifelong exposure history, the exposome complements the genome. The exposome entails the nature and variation of each exposure affecting individuals from conception to death. These non-genetic exposures have been categorized as internal, specific external, and general external (5). Among the specific external exposures, diet, lifestyle factors, occupation, chemical contamination, environmental pollution, radiation, infections, and medical interventions have historically been the focus of major epidemiological studies. Broader social, economic, and psychological factors such as education, socioeconomic status, stress, social capital, urban or rural conditions, and climate. Finally, the internal components impact the cellular environment and include body morphology, physical exercise, metabolism, inflammation, lipid peroxidation, oxidative stress, endogenous circulating hormones, and gut microflora. These three categories overlap, and sometimes, a particular exposure may be categorized in different categories.

However, the influence of the whole exposome on health outcomes is still largely misunderstood and does not amount to the sum of its different individual components. A further difficulty is that the exposome is dynamic and thus difficult to characterize in practice. This would theoretically require sequential measures over a lifetime or fewer measurements characterizing exposure. Although it is far from a complete description of the exposome, some have thus proposed a number of crucial periods where measures of the exposome could be made and, despite the limited measurements, still describe meaningful individual exposure patterns: gestation, early childhood, puberty, and reproductive years (6, 7). Hence, the concept can be translated into a useful tool to better understand human diseases and ways to prevent them. Studying exposomes at the population level may provide a more comprehensive understanding of the environmental factors affecting health outcomes, and it may help identify strategies for health promotion and the prevention of diseases in communities.

French Guiana is a French overseas territory. It lies on the Guiana Shield in South America, between Brazil at the eastern border and Suriname at the western border. This administrative and geographic situation captures the duality of this territory when reflecting on health (Table 1).

**Table 1:** Selected differences between French Guiana and mainland France.

Metric*	French Guiana	Mainland France
General practitioner density (per 100,000 inhabitants)	42	91
Specialist density (per 100,000 inhabitants)	24	87
Dentist density (per 100,000 inhabitants)	27	56
Nurses density (per 100,000 inhabitants)	130	181
Health expenditure *per capita* (Euros)	2,500	4,664
Median age (years)	24	42
Number of hospital beds per 1,000 (medicine)	2.49	2.33
Number of hospital beds per 1,000 (surgery)	0.56	1.20
Life expectancy at birth in male individuals (2019- before COVID, in years)	76.5	79.8
Life expectancy at birth in individuals (2019- before COVID, in years)	82.5	85.6
Total fertility rate	3.6	1.98
Standardized death rate from stroke (per 100,000)	71.5	45.7
HIV new diagnoses per year (per 100,000)	71.1	10
Smoking (% of population aged 15–75 years)	12.2	29.7
Daily alcohol (% of population aged 15–75 years)	5	10
Cannabis use during past year (% of population aged 15–64 years)	8	11

* From (8–11).

There are great variations between population subgroups, notably in poorer countries, leading to substantial inconsistencies with those predicted by the classical epidemiologic transition theory. In this context, using public data, we aimed to determine how the singular case of French Guiana fit and transitioned in the epidemiologic transition framework. The data show a gradual decline in infant mortality to values above 8 per 1,000 live births. Premature mortality rates were greater but declined more rapidly in French Guiana than in mainland France until 2017 when they reascended in a context of political turmoil followed by the COVID-19 pandemic and strong reluctance to get vaccinated. Although infections were a more frequent cause of death in French Guiana, there is a marked decline, and circulatory and metabolic causes are major causes of premature death. Fertility rates remain high (>3 live births per woman), and the age structure of the population is still pyramid-shaped. The singularities of French Guiana (rich country, universal health system, and widespread poverty) explain why its transition does not fit neatly within the usual stages of transition. Beyond gradual improvements in secular trends, the data also suggest that political turmoil and fake news may have detrimentally affected mortality in French Guiana and reversed improving trends (8–11).

As a French territory, the rights to health and the health system are that of continental France, but as a South American territory, much of the environment is akin to that of the Amazon region. Socioeconomically, the gross national product *per capita* of French Guiana is the highest in Latin America. Many immigrants arrive to search for better economic prospects, but, in practice, they often live in great poverty when they reach French Guiana (8). In practice, nearly half of the adult population and 29% of the total population are of foreign origin. In this context, the proportion who lives below the poverty threshold is nearly half of the total population. As a tropical Amazonian territory, French Guiana still has a large variety of infectious diseases; the environment and climate are also quite different from mainland France. We have recently compared life indicators and mortality causes between French Guiana and mainland France, showing that there was generally a 2- to 3-year difference in terms of life expectancy at birth, which presumably reflected differences in mortality rates for different pathologies, with notable differences that were sensitive to primary healthcare and thus preventable (8). Although differences in healthcare may partly explain the observed differences (lack of physicians), other very important explanations are also involved. What seemed apparent was that, from what is presently known about exposure to different risk factors in French Guiana, the typical exposome in French Guiana is not the same as that of mainland France. Furthermore, health inequalities impacting nearly half of adults suggest that global measurements of life expectancy presumably conflate at least two distinct situations: persons who have similar life expectancies as in mainland France and persons living in precariousness who have far greater mortality rates than their wealthier counterparts. The present narrative review thus aimed to synthesize what is known about statistical regularities regarding exposures and sketch typical French Guiana exposomes in relation to health outcomes. This conceptual exercise nevertheless aims to refine the health system’s efforts to alleviate the impact of the main risk factors in French Guiana.

## Methods

A narrative review was conducted to synthesize the available evidence from the territory. The evidence was extracted by MN after a PubMed search between 1990 and 2023 with the following keywords: French Guiana AND (hypertension OR obesity OR diabetes OR hunger OR nutrition OR risk factor OR infectious diseases OR mortality OR pregnancy OR life expectancy OR exposure OR exposome) which yielded 747 results. The abstracts were screened by MN, and additional references can be found in the bibliography. Overall, we selected 63 references to support the narrative review.

From the breadth of the available literature and from local expertise, we attempted to distill the information about the exposome broken down into different meaningful categories to make it easier to grasp. We generated an ordinal scale that is not based on any existing tool; we also created urban/rural or rich/poor distinctions, which resulted from the combination of epidemiological studies and local expert knowledge.

## Results

### Specific external exposures

Since the French first set foot in French Guiana, the burden of infectious diseases has been prominent in the public stereotype of French Guiana as a land of fevers, snakes, and spiders. As biodiversity has a latitudinal gradient, the biodiversity of human and animal pathogens is highest as one approaches the equator. The range of meaningful infectious pathogens is broad, with malaria, Amazonian toxoplasmosis, leishmaniasis, gastrointestinal nematodes, arboviroses, HIV, HTLV-1, HPV, HBV, Syphilis, *Mycobacterium ulcerans*, tuberculosis, leprosy, Q fever, leptospirosis, histoplasmosis, cryptococcosis, and more (8, 12–19). Apart from COVID-19, the infectious pathogen with the greatest burden of disease in the past 40 years has been HIV/AIDS, notably HIV-associated disseminated histoplasmosis, but mortality has considerably dropped (20). Infectious pathogens such as HPV (21), HTLV-1 (12), and *H. pylori* (22) have a substantial impact on infection-mediated cancers. Perhaps one of the major health consequences of infectious pathogens concerns pregnant women and their fetuses (14, 15, 18, 23–25).

Smoking is less frequent in French Guiana than in mainland France (12.2% vs. 29.7%, respectively) (9). For alcohol consumption, 35% of French Guianese report drinking weekly, including 5% of daily drinkers, levels that are well below those in mainland France (48% of weekly drinkers, including 10% of daily drinkers) (9). The major risk factors of tobacco and alcohol are thus markedly less prevalent in French Guiana than in mainland France.

For illegal drug use, experimentation of cannabis was lower in French Guiana than in mainland France (25% vs. 41%, respectively) and twice as often in men (34%) relative to women (17%) (9). Drug use during the last 12 months concerned 8% of the population in French Guiana and 11% in mainland France. For other illicit drugs, the levels of experimentation were also significantly lower in French Guiana: 2% for cocaine vs. 5% in mainland France, 1.8% for hallucinogenic mushrooms vs. 5% in mainland France, 1.3% for ecstasy or MDMA vs. 4% in mainland France, and less than 1% for poppers (vs. 7%), amphetamines (vs. 2%), and crack cocaine (vs. 2%) (9).

Apart from the above lifestyle factors, nutritional problems are particularly worrisome in French Guiana. Despite the lack of precise studies detailing food consumption in different populations (culture and socioeconomic level greatly shape diet), the nutritional transition from predominant undernourishment to predominance of overnourishment is not complete. While overweight and obesity are very frequent (26, 27), it has recently been emphasized that food insecurity and nutritional deficiencies are perhaps even more frequent (28, 29) and often associated with overweight/obese persons with micronutrient deficiencies. These nutritional problems largely overlap with social precariousness—which concerns over half of the population in French Guiana—and affect the vulnerable period of the first 1,000 days after conception (28). The consequences of what happens in this time frame extend far beyond, as studied in the research field of the Developmental Origins of Health and Disease (DOHaD) (30). Beyond the nutritional aspects, food is also associated with the serious hazards of heavy metals such as mercury and lead. Given the importance of illegal gold mining, mercury pollution and concentration by predatory fish is a significant problem with autochthonous populations who often rely on fishing (31–38). There again, the first 1,000 days after conception is a period of particular vulnerability, and some studies in French Guiana identified specific neurological and neuropsychological deficits in Amerindian children that were consistent with known targets of mercury neurotoxicity (34). More recently, the importance of exposure to lead has been revealed, with lead from hunting cartridges or Cassava and other tubers concentrating lead from the soil being suspected, but the sources of such widespread exposure are still being investigated (39, 40). Approximately 25% of pregnant women in western French Guiana displayed blood lead levels of ≥50 μg/L and 5% ≥100 μg/L. There again, the poorest were more at risk, and the highly vulnerable first 1,000 days of life were particularly exposed. Despite progress, safe drinkable water remains a problem in some socially disfavored parts of French Guiana (41).

In the context of widespread poverty, informal occupations are also associated with a broad range of risks: for gold miners, severe nutritional problems (42), mercury poisoning, infectious diseases, and external causes such as accidents or homicides. (19, 43–47); for sex workers or boatmen, a greater risk of HIV and Sexually transmitted infections (48–50).

### General external exposures

Fifty-three percent of the population in French Guiana lives under the poverty level, and 29% live in great poverty (51). At the hospital, a cross-sectional study found that 75% of patients were socially precarious (52). Overall, in the 16–65 years group, 2 of every 5 individuals have severe difficulties with written language (53). There is a large overlap between precariousness and being foreign in French Guiana. Overall, 37% of the total population is of foreign nationality (and about half of adults), which is more than what has been reported anywhere else (54). By contrast, in mainland France, 7.6% of the population is foreign. Overall, between 15 and 18% of the population is undocumented in French Guiana vs. between 0.89 and 1.19% in mainland France (55). In 2015, births from foreign mothers were estimated to have outnumbered births from French mothers. Thus, 60% of babies were born from a foreign mother, and 40% were born from a foreign father, mainly Haitian. In 2017, Haitian mothers delivered a quarter of babies born in French Guiana. The overall number of newborns with only one French parent was increasing and, in 2017, represented 38% of births (56, 57). According to the French Office for the Protection of Refugees and Stateless Persons, 7,934 adult asylum seekers were registered in French Guiana between 2005 and 2014.

Historically, immigration has been a major driver of the social fabric of French Guiana. This sensitive topic commonly leads to the mistaken assumption that immigrants come to French Guiana for health reasons. However, studies refute this claim and suggest that only 1% of immigrants came to French Guiana for health reasons (58). Perhaps an exception concerns deliveries in western French Guiana, where women from Suriname cross over to deliver in the maternity ward of Saint Laurent du Maroni hospital. Although foreigners may sometimes suffer from discrimination (59, 60), they also benefit from extra efforts to correct social inequalities in health (52, 61, 62). However, because they do not know their rights or because complex administrative requirements discourage them from pursuing them, immigrants often do not apply for social benefits that they would be entitled to, thus contradicting common preconceptions. Thus, in 2015, 82% of “undocumented” people who had arrived for more than 3 months were not covered by the Aide Médicale Etat health insurance (health insurance for undocumented foreigners) despite being eligible for it (63). As a general rule of thumb, for infectious, chronic, obstetrical, or nutritional diseases, immigrants tend to have more advanced pathologies, which occur at younger ages (60, 64–69). However, for prolonged diseases, once immigrants benefit from health insurance, the differences between foreign and French or between precarious and non-precarious persons tend to disappear because the health system strives to compensate for their vulnerabilities by enhancing therapeutic education, health mediation and translation services, nurse visits at home (62), and an increased tendency to hospitalize patients living in difficult conditions (61). A complementary explanation is that, with time, immigrants develop social capital and expertise in the health system. Psychosocial stress and trauma linked to migration and poverty are widespread, notably among immigrants from countries that have undergone natural disasters and/or political violence and who have, at times, been preyed upon by human traffickers during their voyage toward French Guiana. Preliminary results from a World Health Organization study have shown that 45.2% of persons in French Guiana had experienced traumatic events vs. 30.2% in mainland France, and 7.8% experienced post-traumatic stress disorder in French Guiana vs. 0.7% in mainland France.

Finally, 20% of the population in French Guiana resides in the interior, in villages that are mostly only accessible by canoe or air, with only access to primary care (52). Regarding the access or use of the health system, there are complex differences between communities, which pertain to social conditions but also to cultural factors. Hence, despite the free availability of the COVID-19 vaccine, despite health promotion efforts, cultural mediation, translation, and outreach campaigns, French Guiana has the lowest vaccination rate in France and Latin America. This apparently is a major social fact that aligns with ethnic communities and illustrates the strong distrust and resentment toward the Western health system (70–72). It is not the only visible social fact on the yearly life statistics: In 2017, a prolonged social movement (all major roads were blocked for a month, and Cayenne Hospital was struck by a 74-day strike) demanding strong measures to fill the perceived structural gap between mainland France and French Guiana, notably for healthcare, was synchronous with the reascension of premature mortality (8). Although causal pathways can only be hypothesized, “self-destructive” peaks of social opposition hence seem to have sufficiently large effects to be visible on life statistics—i.e., a 3- to 4-year drop in life expectancy at birth in French Guiana vs. half a year in France. A simple yet plausible explanation is that, in a territory with a fragile health system and a large fragile population, hampering access to care to make a point is not such a good idea, broadcasting messages emphasizing the dangerousness of the health system and embarking the population on misguided solutions for health problems to make political points have dramatic consequences.

### Internal exposures

With over half of the population affected in French Guiana, overweight and obesity are more frequent than in mainland France, notably among women, precarious persons, and those with low educational levels. Women were much more often affected by obesity than men: 23% vs. 13%, whereas in mainland France, the proportion of obese in the same national study found that 12% of men and 12% of women were obese, and another study found obesity prevalences of 15.8% for men and 15.6% for women (9, 11).

In 2014, physical activity was on par with that of mainland France in those aged less than 60 years. However, physical exercise was substantially lower than in mainland France in those aged over 60 years (32% vs. 18% of the 61–75 years group, respectively). Men exercised more than women in the population in general (44% vs. 28%, respectively) (9). A more recent estimate (2022–2021) found that, overall, the proportion of the population exercising at least 30 min per day was lower in French Guiana than in mainland France, both in men (29% vs. 36%, respectively) and in women (19% vs. 27%, respectively) (11).

In this context, the standardized prevalence of treated diabetes is greater than in mainland France: 7.7% compared with 4.6%. Despite the youthfulness of the population in French Guiana (median age 23 years), arterial hypertension is a major problem, notably among women (73). Thus, hypertension constitutes the major risk factor associated with strokes, myocardial infarction, and end-stage renal disease (65, 74, 75). Lipid abnormalities are common, but a recent study of Lpa among people with diabetes showed that Lipoprotein (a) (Lp(a)) concentrations, in contrast with mainland France, did not have the same prognostic value, suggesting that lipoprotein polymorphisms make it difficult to compare their prognostic values between populations with different ancestries (76).

### Synthetic comparisons

Table 2 shows a simplified view of the main components of the exposomes of French Guiana and mainland France. Table 3 then compares the main components of the exposomes for the poorer half and richer half in French Guiana. Finally, Table 4 compares the main components of the exposomes between rural and urban French Guiana. Regarding nutritional deficiencies, the differences shown in Table 4 are complex: in the interior, the diversity is poorer than in urban areas, given supply difficulties; however, given the possibilities of fishing and cultivating, food insecurity tends to be lower, but the risk of heavy metals is far greater. In urban areas, the problem is not linked to the irregular supply of shops but to poverty.

**Table 2:** Comparison of the main differences in exposures between French Guiana and mainland France.

Specific external exposures*			General external exposures*			Internal exposures*		
Factor	French Guiana	Mainland France	Factor	French Guiana	Mainland France	Factor	French Guiana	Mainland France
Access to healthcare	++	+++	Poverty	+++	+	Obesity	+++	++
Trust in healthcare	+	+++	Illiteracy	+++	+	Diabetes	+++	+
Infectious diseases	+++	+	Hard to reach areas	+++	+	Lipid anomalies	+	+++
Caloric excess	+++	++				Physical exercise	+	++
Nutritional deficiencies	+++	−				Hypertension	+++	+
Food insecurity	+++	−						
Food borne heavy metals	+++	−						
Smoking	+	+++						
Alcohol	+	+++						
Drugs	+	++						
Work-related hazard	+++	−						
Accidents	+++	+						

* The color codes used for the synthetic comparisons are meant to reinforce the ordinal scale ranging from – to +++. They range from green (the lowest risk) to yellow, orange, and red (the greatest risk).

**Table 3:** Comparison of the main differences in exposures between the poorer and richer halves of French Guiana.

Specific external exposures*			General external exposures*			Internal exposures*		
Factor	Poorer half	Richer half	Factor	Poorer half	Richer half	Factor	Poorer half	Richer half
Access to healthcare	+	+++	Poverty	+++	−	Obesity	+++	++
Trust in healthcare	++	+	Illiteracy	+++	−	Diabetes	+++	++
Infectious diseases	+++	+	Geographic isolation	+++	−	Lipid anomalies	+	+
Caloric excess	+++	++				Physical excercise	+	+++
Nutritional deficiencies	+++	−				Hypertension	+++	+++
Food insecurity	+++	−						
Food borne heavy metals	+++	−						
Smoking	+	+						
Alcohol	+	+						
Drugs	+	+						
Work-related hazard	+++	+						

* The color codes used for the synthetic comparisons are meant to reinforce the ordinal scale ranging from – to +++. They range from green (the lowest risk) to yellow, orange, and red (the greatest risk).

**Table 4:** Comparison of the main differences in exposures between urban vs. rural French Guiana.

Specific external exposures*			General external exposures*			Internal exposures*		
Factor	Urban	Rural	Factor	Urban	Rural	Factor	Urban	Rural
Access to primary healthcare	+	++	Poverty	++	+++	Obesity	+++	+
Trust in healthcare	+	++	Illiteracy	+	++	Diabetes	+++	+
Infectious diseases	+++	+	Geographic isolation	−	+++	Lipid anomalies	+	+
Caloric excess	+++	++				Physical excercise	+	+++
Nutritional deficiencies	+++	++				Hypertension	+++	++
Food insecurity	+++	+						
Food borne heavy metals	−	+++						
Smoking	+	+						
Alcohol	+	++						
Drugs	+	+						
Work-related hazard	+++	++						
Accidents	+++	++						

* The color codes used for the synthetic comparisons are meant to reinforce the ordinal scale ranging from – to +++. They range from green (the lowest risk) to yellow, orange, and red (the greatest risk).

**Figure 1:**
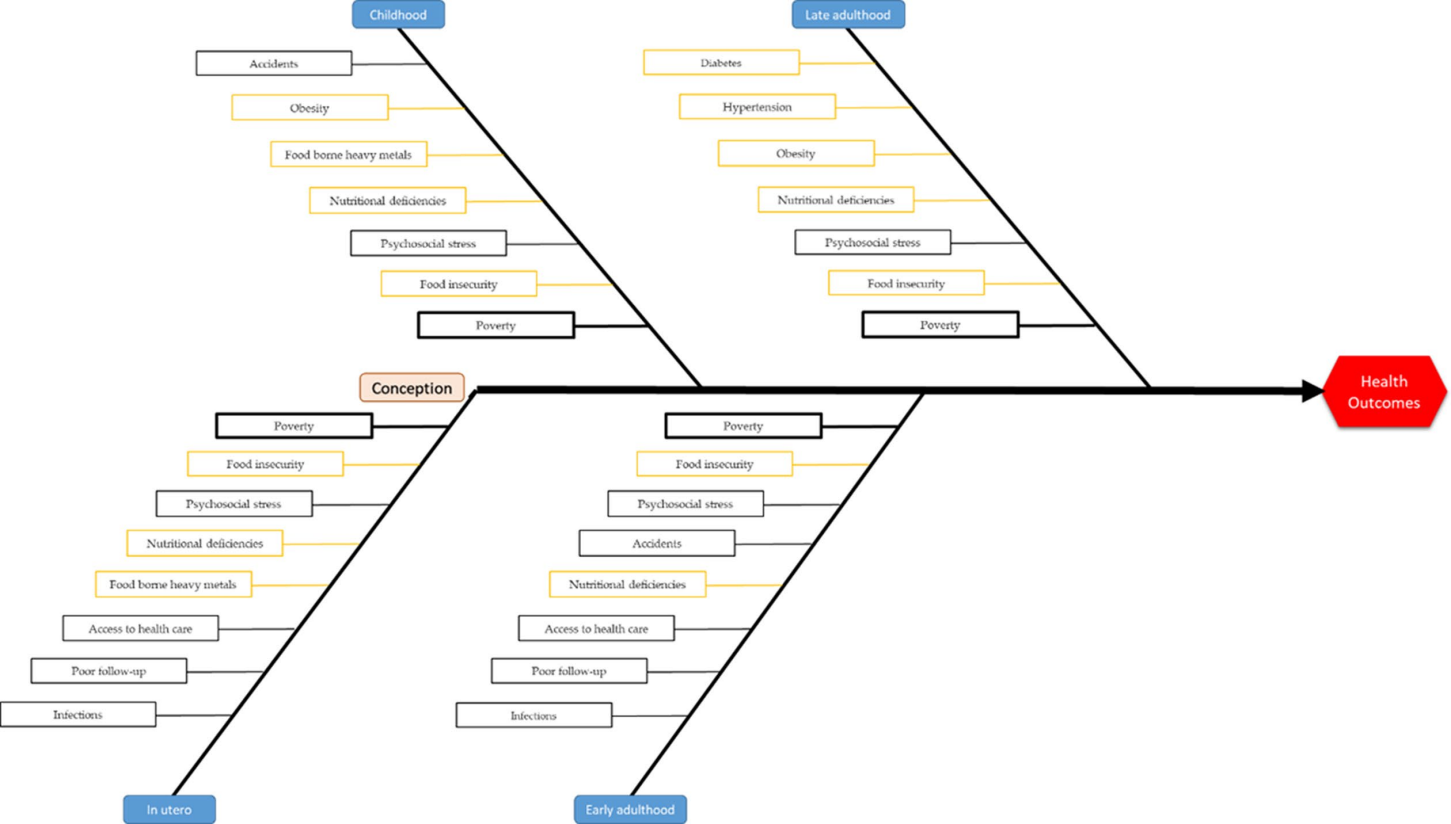
Synthetic representation of the main exposures at different stages of life.

Figure 1 shows a Fishbone graph representation of the main exposures at different stages of life (*in utero*, childhood, and early and late adulthood) in French Guiana. The figure shows that the first 1,000 days since conception are potentially at risk for a broad range of risks. The figure also shows (in orange) that the security of the food chain is at the origin of many deleterious health consequences. Boxes in bold emphasize the role of poverty at all stages of life. Early adulthood is considered to be adults of less than 46 years, and late adulthood is considered to be adults of ages >45 years.

## Discussion

### Statistical regularities and target populations

In public health, problems can be approached in three different ways: An approach focused on a specific problem (i.e., cancer, stroke, HIV, etc.), an approach focused on the determinants of health, and finally an approach focused on a specific population. Here, whether looking at internal, specific, or general external exposures, two main observations can be made: first, as far as we can infer the typical exposome in French Guiana, there are great differences with mainland France, which makes the interpretation of differences in life statistics difficult; second, it is apparent that social inequalities in French Guiana are so massive that the metrologic significance of life statistics, or standardized incidences of specific causes of death from death certificates, seems to be a crude averaging of very different worlds with very different exposomes. For example, we had observed among a cohort of women with breast cancer that in French Guiana, there was a 7% deficit in 5-year survival relative to mainland France. However, when stratifying by country of residence, there was no longer any difference for French citizens, whereas foreign-born women had a 24% deficit in survival at 5 years (77). Hence, given the great territorial contrasts, crude measures are often misleading. It is quite plausible to hypothesize that, in French Guiana, the non-precarious half of the population has a similar life expectancy to those living in mainland France. But with a median income in French Guiana only half that of France (920 Euros vs. 1837 Euros, respectively), and with food prices 34% greater in French Guiana, large differences in life statistics are expected. We used the INSEE’s tool to estimate life expectancy at birth stratified by income, and there was a 5.8-year difference for men and a 3.8-year difference for women between the 1,000 euro per month group and the 2000 euro per month group (78). Because 23% of persons in French Guiana live with less than 550 euros per month, estimations of the difference in life expectancy at birth were computed between those living with 500 euros per month vs. those living with 2000 euros per month. Hence, there was a 9.3-year difference for men and a 6.6-year difference for women. However, these approximations illustrating the impact of poverty may be interesting, but they use national tools calibrated using national probabilities of survival at different ages, which do not take into account the striking differences in the exposomes of French Guiana and mainland France. Another meaningful way to stratify the population could be to distinguish the rural populations in the interior from those in the coastal and mostly urban areas. We presently lack data from the most remote villages for robust comparisons. Aiming to get the “big picture,” we synthesized the available data and local expert knowledge. Although we assembled much expertise and data, we wish to acknowledge that each of our assumptions in Tables 2–4 can be criticized. Despite these limitations, the present study is the first to have such a holistic aim.

Perhaps the most striking fact this panorama shows is that being a fetus or a young child in French Guiana is fraught with multiple threats. The 2.6-fold increase in infant mortality relative to mainland France and the recent increase in causes of death from congenital anomalies or chromosomal causes may not be so surprising in this context. In French Guiana, poverty and poor pregnancy follow-up; renouncing to healthcare; wide variety of infectious diseases; very high prevalence of food insecurity, psychosocial stress, micronutrient deficiencies, and obesity and metabolic problems; and frequent exposure to lead and mercury in rural areas constitute a stunningly challenging environment for a new human being to develop into. Beyond an assemblage of causal explanations built on epidemiological observations, the present review attempts to integrate the available narrow epidemiologic data into a broader holistic view (79).

### From concept to practical interventions to improve health

The national and international project on the first 1,000 days of life emphasizes the crucial importance of this period. The number of potentially harmful exposures faced by fetuses and young infants emphasizes that our first priority should be to actively endeavor to alleviate these risks through proactive measures toward improving follow-up of pregnant women and prevention of food insecurity and nutritional deficiencies, toxic exposures, infectious diseases, and psychosocial risks. In a nutshell, this requires a population-based approach targeting the most socially vulnerable pregnant women and projecting a variety of human resources toward those who need them the most. A second population, which includes the first population, is that of the socially precarious in general, whether they are urban immigrants or rural autochthonous populations. Given the accumulation of risk factors among the socially precarious, proactive actions seem crucial to reduce the incidence of the major causes of premature death. Although the conclusions of this attempt to reconstitute the major population exposomes could seem futile—we already know all that!—it pleads for integrated and more strategic approaches if one aims to improve human health for most who live in French Guiana. While the genome results from a long evolutionary history and is beyond the grasp of public health, reshaping the exposome is indeed the major field of public health efforts. In French Guiana, the multiethnic population entails genomes shaped by very different ancestral trajectories with differences in potential vulnerabilities (i.e., Amerindians for type 2 diabetes, obesity, or certain mental health issues; those with African ancestry for sickle cell anemia, obesity, hypertension, type 2 diabetes, or prostate cancer). Although there is not much we can do about the genes themselves, the population exposome view offers a strategic view for public health action to alleviate the impact of exposures that lead to most of the burden of disease behind premature mortality. We emphasize that this view does not imply that the risk for different health outcomes can neatly be broken down by adding the attributable fractions of all genetic and environmental exposure risks, adding up to a total of 100%: they do not (80). While occasional cases of marasmus and kwashiorkor have disappeared in the past two decades, a striking fact is how a substantial part of the population’s health is affected by its sources of nutrition: often too many calories in relation to one’s activity, but also often not enough food and too few micronutrients, and even a risk of heavy metal poisoning. This affects the health system but has ramifications far beyond medical conditions; it also ramifies education (children with nutritional deficiencies or lead poisoning have learning difficulties), those dealing with road infrastructure, the police (road traffic accidents), the justice system (lead poisoning has been associated with crime levels), agriculture, and commerce, to name only a few. This diagnosis, in French territory, should elicit a strong coordinated response by all sectors of society that ensure a steady supply of safe quality food, with a particular focus on pregnant women and children. Finally, beyond the confines of French Guiana, on the Guiana Shield and in the Amazon, the exposome profiles are likely to share many features of what we have reconstructed here, and therefore, public health actors in the region would benefit from sharing knowledge and experiences in this complex context.

## Author contributions

MN: first draft and final draft writing. CB, MD, YL, CR, CM, RG, AA, MG, KA, NS, VL, LE, RS, FT, CJ, and NH: review and editing. All authors contributed to the article and approved the submitted version.
